# Emerging Deep Cutaneous Fungal Infection Caused by *Cyphellophora* Species in a Diabetic Patient

**DOI:** 10.1002/kjm2.70204

**Published:** 2026-03-16

**Authors:** Yi‐Shan Teng, Pei‐Lun Sun, Cheng‐Che E. Lan

**Affiliations:** ^1^ Department of Dermatology, Kaohsiung Medical University Hospital Kaohsiung Medical University Kaohsiung Taiwan; ^2^ Department of Dermatology, College of Medicine Kaohsiung Medical University Kaohsiung Taiwan; ^3^ Research Laboratory of Medical Mycology, Chang Gung Memorial Hospital, Linkou Medical Centre Taoyuan Taiwan; ^4^ School of Medicine Chang Gung University Taoyuan Taiwan; ^5^ Department of Dermatology, Chang Gung Memorial Hospital, Linkou Medical Centre Taoyuan Taiwan

To the Editor,

1

A 55‐year‐old woman with poorly controlled diabetes mellitus and a history of frequent steroid injections for muscle soreness presented with a 10‐month history of pruritic, erythematous, indurated plaques resembling keloids on both forearms (Figure 1A). These lesions were unresponsive to steroid and antihistamine. She denied recent trauma, surgeries, infections, travel, or outdoor activities and exhibited no systemic symptoms. Laboratory investigations excluded bacterial, viral, parasitic, and mycobacterial infections. A skin biopsy showed granulomatous inflammation containing faintly pigmented spores (Figure 1D). Grocott methenamine silver (GMS) and periodic acid–Schiff (PAS) stains highlighted fungal hyphae and spores within the deep dermis (Figure 1E,F), supporting a diagnosis of deep cutaneous fungal infection (DCFI). Microscopy demonstrated branching, irregularly septated hyphae (Figure 1G,H). Fungal culture on potato dextrose agar (PDA) yielded a pigmented fungus (Figure 1C) that failed to sporulate, precluding definitive morphological identification. Internal transcribed spacer (ITS) sequencing showed 92% similarity to *Cyphellophora* pluriseptata (CBS 286.85), confirming DCFI caused by a *Cyphellophora* species. After a 2‐month course of systemic terbinafine, the keloid‐like plaques markedly flattened with partial resolution of erythema (Figure 1B).

**FIGURE 1:**
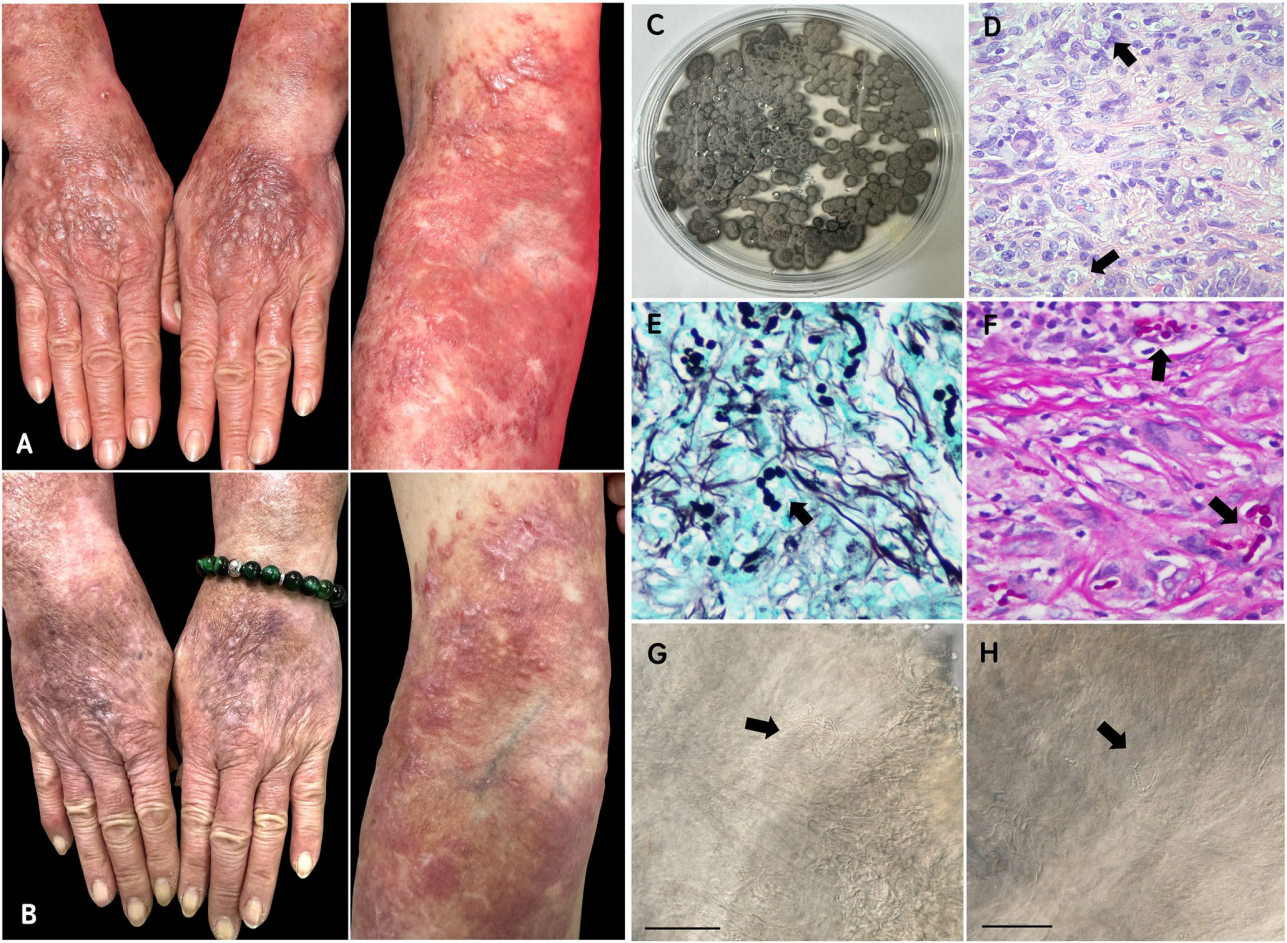
Clinical and histopathological features of a deep cutaneous fungal infection due to *Cyphellophora* species, with culture characteristics of the isolate from this patient. (A) Multiple ill‐defined erythematous patches and indurated plaques on the forearms and dorsal hands. Keloid‐like lesions developing on erythematous indurated plaques on the forearm. (B) Significant flattening of the keloid‐like lesions and partial resolution of erythematous areas observed after a 2‐month course of systemic antifungal therapy. (C) Dark gray mold growth observed on Potato Dextrose Agar at 25°C. (D) Slightly pigmented fungi (arrows) visible in hematoxylin–eosin (HE)‐stained sections (original magnification ×400). (E) Grocott's methenamine silver (GMS) staining and (F) Periodic acid‐Schiff (PAS) staining highlighted fungal hyphae and spores located in the deep dermis (original magnification ×400). Microscopic examination showed branching irregularly septated hyphae on (G) and (H) (Scale bars = 50 μm).

DCFIs are uncommon infections primarily affecting immunocompromised individuals, such as those with poorly controlled diabetes or on immunosuppressive therapy. They present varied clinical manifestations, including plaques, nodules, and ulcers, and pose diagnostic challenges. If untreated, DCFIs can cause severe complications or mortality. *Cyphellophora*, a genus of black yeast‐like fungi, has emerged as a notable cause of superficial cutaneous mycoses [1] and DCFIs [2]. These fungi, characterized by multiseptated, curved conidia, thrive in diverse environments such as soil, plants, and moist household surfaces [3, 4]. This ecological versatility suggests that infections may arise from everyday exposures, even without outdoor activities or direct environmental contact. In this case, the patient's diabetes and frequent steroid use likely predisposed her to infection, emphasizing the role of host factors in DCFI pathogenesis. Traditional diagnostic methods, including culture and histopathology, often fail to identify nonsporulating or rare fungal pathogens. Molecular diagnostics, such as ITS sequencing, are critical in such cases. The fungal isolate in this patient was identified only at the genus level, highlighting the need for further taxonomic studies to determine if it represents a novel *Cyphellophora* species. Management of DCFIs requires prolonged antifungal therapy, guided by susceptibility testing. Studies have demonstrated that azoles and terbinafine are effective against *Cyphellophora* species [5]. Our case reinforces their efficacy, with terbinafine leading to clinical improvement.

In conclusion, this report underscores the emerging role of *Cyphellophora* as an opportunistic pathogen in immunocompromised patients and highlights the indispensable role of molecular diagnostics in identifying rare fungi. The ecological adaptability of *Cyphellophora* indicates that damp environments may contribute to infection risk, underscoring the importance of hygiene and minimizing exposure to such areas. Further research is necessary to expand our understanding of the clinical spectrum, pathogenesis, and optimal management strategies for *Cyphellophora* infections.

## Conflicts of Interest

The authors declare no conflicts of interest.

## Data Availability

The data that support the findings of this study are available from the corresponding author upon reasonable request.
